# 1-Methyl-3-*n*-tetra­decyl­imidazolium bromide monohydrate

**DOI:** 10.1107/S160053680903935X

**Published:** 2009-10-03

**Authors:** Yu Chen, Wei Song, Juan Xu, Xiao-Rong Yang, Dan-Bi Tian

**Affiliations:** a Department of Applied Chemistry, College of Science, Nanjing University of Technology, Nanjing 210009, People’s Republic of China

## Abstract

In the title ionic liquid salt hydrate, C_18_H_35_N_2_^+^·Br^−^·H_2_O, the side chain in the cation has an extended conformation. The crystal structure is stabilized primarily by O—H⋯Br hydrogen bonds. C—H⋯O and C—H⋯Br inter­actions are also present.

## Related literature

For background to imidazolium ionic liquids, see: Ding *et al.* (2007 ▶, 2008 ▶).
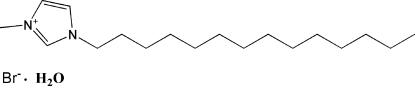


## Experimental

### 

#### Crystal data

C_18_H_35_N_2_^+^·Br^−^·H_2_O
*M* *_r_* = 377.41
Triclinic, 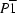

*a* = 5.5130 (11) Å
*b* = 7.8390 (16) Å
*c* = 25.114 (5) Å
α = 94.74 (3)°
β = 94.45 (3)°
γ = 102.06 (3)°
*V* = 1052.7 (4) Å^3^
*Z* = 2
Mo *K*α radiation
μ = 1.96 mm^−1^
*T* = 298 K
0.20 × 0.10 × 0.10 mm

#### Data collection

Enraf–Nonius CAD-4 diffractometer
Absorption correction: ψ scan (North *et al.*, 1968 ▶) *T*_min_ = 0.696, *T*_max_ = 0.828
4277 measured reflections
3843 independent reflections
2593 reflections with *I* > 2σ(*I*)
*R*_int_ = 0.030
3 standard reflections every 200 reflections intensity decay: 1%

#### Refinement

*R*[*F* ^2^ > 2σ(*F* ^2^)] = 0.056
*wR*(*F* ^2^) = 0.163
*S* = 1.01
3843 reflections
199 parameters
H-atom parameters constrained
Δρ_max_ = 0.39 e Å^−3^
Δρ_min_ = −0.29 e Å^−3^

### 

Data collection: *CAD-4 Software* (Enraf–Nonius, 1989 ▶); cell refinement: *CAD-4 Software*; data reduction: *XCAD4* (Harms & Wocadlo, 1995 ▶); program(s) used to solve structure: *SHELXS97* (Sheldrick, 2008 ▶); program(s) used to refine structure: *SHELXL97* (Sheldrick, 2008 ▶); molecular graphics: *SHELXTL* (Sheldrick, 2008 ▶); software used to prepare material for publication: *SHELXTL*.

## Supplementary Material

Crystal structure: contains datablocks I, global. DOI: 10.1107/S160053680903935X/tk2543sup1.cif

Structure factors: contains datablocks I. DOI: 10.1107/S160053680903935X/tk2543Isup2.hkl

Additional supplementary materials:  crystallographic information; 3D view; checkCIF report

## Figures and Tables

**Table 1:** Hydrogen-bond geometry (Å, °)

*D*—H⋯*A*	*D*—H	H⋯*A*	*D*⋯*A*	*D*—H⋯*A*
O*W*—H*WA*⋯Br	0.85	2.57	3.397 (5)	165
O*W*—H*WB*⋯Br^i^	0.85	2.61	3.434 (5)	163
C15—H15*A*⋯Br^ii^	0.93	2.75	3.659 (5)	166
C17—H17*A*⋯O*W*	0.93	2.36	3.217 (7)	153

Symmetry codes: (i) 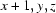; (ii) 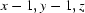.

**Fig. 1.**
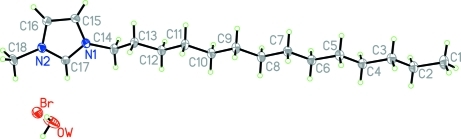
Molecular structure of (I) showing the atom-numbering scheme. Displacement ellipsoids are drawn at the 50% probability level.

## supplementary crystallographic information

### Comment

The title compound (I) is an example of am imidazolium ionic liquid, which has an influence upon the plasticization and crystallization of polypropylene (Ding *et al.*,  2007 & 2008). Herein, the crystal structure of (I) is described.

The tetradecyl side-side chain in cation, Fig. 1, has an extended conformation. The crystal structure features O-H···Br hydrogen bonding and is further stabilised by C-H···O and C-H···Br interactions, Table 1.

### Experimental

Compound (I) was prepared following a modified literature procedure (Ding *et al.*, 2007). 1-Methylimidazole (8.21 g, 0.1 mol) was mixed with tetradecyl bromide (27.7 g, 0.1 mol) in toluene (150 ml) and refluxed for 24 h. The mixture was subsequently cooled to room temperature and filtered. The solids were washed several times with ethyl acetate (600 ml) and the product dried in vacuum (yield: 33.36 g, 92.8%). Colourless crystals were obtained by evaporating a chloroform solution slowly at room temperature for about 5 days.

### Refinement

The H atoms were geometrically placed (O-H = 0.85 Å and C-H = 0.93-0.97Å) and refined as riding with U_iso_(H) = 1.2U_eq_(C) or 1.5U_eq_(O, methyl-C).

### Crystal data

**Table:**

C_18_H_35_N_2_^+^·Br^−^·H_2_O	*Z* = 2
*M**_r_* = 377.41	*F*(000) = 404
Triclinic, *P*1	*D*_x_ = 1.191 Mg m^−^^3^
Hall symbol: -P 1	Melting point: 327 K
*a* = 5.5130 (11) Å	Mo *K*α radiation, λ = 0.71073 Å
*b* = 7.8390 (16) Å	Cell parameters from 25 reflections
*c* = 25.114 (5) Å	θ = 10–13°
α = 94.74 (3)°	µ = 1.96 mm^−^^1^
β = 94.45 (3)°	*T* = 298 K
γ = 102.06 (3)°	Square, colourless
*V* = 1052.7 (4) Å^3^	0.20 × 0.10 × 0.10 mm

### Data collection

**Table:**

Enraf–Nonius CAD-4 diffractometer	2593 reflections with *I* > 2σ(*I*)
Radiation source: fine-focus sealed tube	*R*_int_ = 0.030
graphite	θ_max_ = 25.3°, θ_min_ = 1.6°
ω/2θ scans	*h* = 0→6
Absorption correction: ψ scan (North *et al.*, 1968)	*k* = −9→9
*T*_min_ = 0.696, *T*_max_ = 0.828	*l* = −30→30
4277 measured reflections	3 standard reflections every 200 reflections
3843 independent reflections	intensity decay: 1%

### Refinement

**Table:**

Refinement on *F*^2^	Primary atom site location: structure-invariant direct methods
Least-squares matrix: full	Secondary atom site location: difference Fourier map
*R*[*F*^2^ > 2σ(*F*^2^)] = 0.056	Hydrogen site location: inferred from neighbouring sites
*wR*(*F*^2^) = 0.163	H-atom parameters constrained
*S* = 1.01	*w* = 1/[σ^2^(*F*_o_^2^) + (0.095*P*)^2^] where *P* = (*F*_o_^2^ + 2*F*_c_^2^)/3
3843 reflections	(Δ/σ)_max_ = 0.001
199 parameters	Δρ_max_ = 0.39 e Å^−^^3^
0 restraints	Δρ_min_ = −0.29 e Å^−^^3^

### Special details

**Table:**

Geometry. All e.s.d.'s (except the e.s.d. in the dihedral angle between two l.s. planes) are estimated using the full covariance matrix. The cell e.s.d.'s are taken into account individually in the estimation of e.s.d.'s in distances, angles and torsion angles; correlations between e.s.d.'s in cell parameters are only used when they are defined by crystal symmetry. An approximate (isotropic) treatment of cell e.s.d.'s is used for estimating e.s.d.'s involving l.s. planes.
Refinement. Refinement of *F*^2^ against ALL reflections. The weighted *R*-factor *wR* and goodness of fit *S* are based on *F*^2^, conventional *R*-factors *R* are based on *F*, with *F* set to zero for negative *F*^2^. The threshold expression of *F*^2^ > σ(*F*^2^) is used only for calculating *R*-factors(gt) *etc*. and is not relevant to the choice of reflections for refinement. *R*-factors based on *F*^2^ are statistically about twice as large as those based on *F*, and *R*- factors based on ALL data will be even larger.

### Fractional atomic coordinates and isotropic or equivalent isotropic displacement parameters (Å^2^)

**Table:**

	*x*	*y*	*z*	*U*_iso_*/*U*_eq_	
Br	0.64859 (10)	0.68030 (7)	0.39439 (2)	0.0596 (2)	
OW	1.0582 (9)	0.4268 (6)	0.3636 (2)	0.116 (2)	
HWA	0.9526	0.4793	0.3766	0.140*	
HWB	1.1957	0.5013	0.3654	0.140*	
N1	0.3634 (7)	0.0580 (5)	0.37818 (15)	0.0463 (9)	
C1	−1.5342 (12)	−0.4153 (9)	−0.2427 (2)	0.0812 (18)	
H1A	−1.5376	−0.4690	−0.2786	0.122*	
H1B	−1.5403	−0.2942	−0.2437	0.122*	
H1C	−1.6754	−0.4744	−0.2262	0.122*	
N2	0.5578 (7)	0.2140 (5)	0.44888 (16)	0.0492 (10)	
C2	−1.2969 (10)	−0.4287 (8)	−0.2104 (2)	0.0638 (14)	
H2A	−1.2905	−0.5515	−0.2107	0.077*	
H2B	−1.1560	−0.3711	−0.2279	0.077*	
C3	−1.2698 (10)	−0.3493 (7)	−0.1529 (2)	0.0562 (13)	
H3A	−1.4101	−0.4077	−0.1354	0.067*	
H3B	−1.2782	−0.2269	−0.1527	0.067*	
C4	−1.0317 (9)	−0.3604 (7)	−0.1205 (2)	0.0547 (13)	
H4A	−0.8911	−0.3028	−0.1381	0.066*	
H4B	−1.0238	−0.4828	−0.1203	0.066*	
C5	−1.0055 (10)	−0.2788 (6)	−0.0629 (2)	0.0541 (12)	
H5A	−1.0110	−0.1560	−0.0631	0.065*	
H5B	−1.1472	−0.3353	−0.0454	0.065*	
C6	−0.7687 (9)	−0.2923 (7)	−0.0303 (2)	0.0531 (12)	
H6A	−0.6270	−0.2353	−0.0477	0.064*	
H6B	−0.7629	−0.4151	−0.0303	0.064*	
C7	−0.7432 (9)	−0.2110 (7)	0.02749 (19)	0.0525 (12)	
H7A	−0.7520	−0.0887	0.0275	0.063*	
H7B	−0.8830	−0.2695	0.0452	0.063*	
C8	−0.5040 (9)	−0.2219 (7)	0.05954 (19)	0.0538 (12)	
H8A	−0.3643	−0.1626	0.0420	0.065*	
H8B	−0.4945	−0.3442	0.0592	0.065*	
C9	−0.4777 (9)	−0.1422 (7)	0.11764 (19)	0.0527 (12)	
H9A	−0.4854	−0.0197	0.1181	0.063*	
H9B	−0.6177	−0.2009	0.1352	0.063*	
C10	−0.2402 (9)	−0.1551 (7)	0.1492 (2)	0.0538 (12)	
H10A	−0.1005	−0.0965	0.1314	0.065*	
H10B	−0.2326	−0.2778	0.1485	0.065*	
C11	−0.2109 (9)	−0.0767 (7)	0.20710 (19)	0.0523 (12)	
H11A	−0.3492	−0.1362	0.2250	0.063*	
H11B	−0.2199	0.0457	0.2079	0.063*	
C12	0.0312 (9)	−0.0890 (7)	0.23820 (19)	0.0513 (12)	
H12A	0.0378	−0.2117	0.2382	0.062*	
H12B	0.1691	−0.0326	0.2196	0.062*	
C13	0.0672 (9)	−0.0071 (7)	0.29559 (19)	0.0500 (12)	
H13A	0.0647	0.1164	0.2962	0.060*	
H13B	−0.0687	−0.0630	0.3148	0.060*	
C14	0.3099 (9)	−0.0271 (7)	0.3228 (2)	0.0548 (13)	
H14A	0.3076	−0.1510	0.3232	0.066*	
H14B	0.4434	0.0225	0.3019	0.066*	
C15	0.2147 (9)	0.0269 (7)	0.4191 (2)	0.0547 (13)	
H15A	0.0581	−0.0476	0.4166	0.066*	
C16	0.3379 (9)	0.1246 (6)	0.4637 (2)	0.0535 (12)	
H16A	0.2834	0.1299	0.4978	0.064*	
C17	0.5686 (9)	0.1733 (6)	0.39745 (19)	0.0513 (12)	
H17A	0.6993	0.2183	0.3778	0.062*	
C18	0.7512 (10)	0.3378 (7)	0.4850 (2)	0.0640 (15)	
H18A	0.8889	0.3829	0.4653	0.096*	
H18B	0.8076	0.2782	0.5138	0.096*	
H18C	0.6829	0.4329	0.4993	0.096*	

### Atomic displacement parameters (Å^2^)

**Table:**

	*U*^11^	*U*^22^	*U*^33^	*U*^12^	*U*^13^	*U*^23^
Br	0.0485 (3)	0.0643 (4)	0.0633 (4)	0.0083 (2)	0.0006 (2)	0.0054 (2)
OW	0.070 (3)	0.073 (3)	0.201 (6)	0.000 (2)	0.043 (3)	−0.010 (3)
N1	0.039 (2)	0.049 (2)	0.049 (2)	0.0097 (18)	−0.0032 (19)	0.0033 (18)
C1	0.077 (4)	0.103 (5)	0.059 (4)	0.023 (4)	−0.014 (3)	−0.010 (3)
N2	0.047 (2)	0.050 (2)	0.047 (3)	0.0039 (18)	−0.0071 (19)	0.0045 (18)
C2	0.059 (3)	0.074 (4)	0.057 (3)	0.020 (3)	−0.002 (3)	−0.005 (3)
C3	0.058 (3)	0.061 (3)	0.050 (3)	0.020 (3)	0.003 (3)	−0.004 (2)
C4	0.053 (3)	0.060 (3)	0.052 (3)	0.017 (2)	0.001 (2)	−0.001 (2)
C5	0.055 (3)	0.054 (3)	0.053 (3)	0.015 (2)	−0.001 (2)	0.000 (2)
C6	0.047 (3)	0.062 (3)	0.051 (3)	0.018 (2)	0.002 (2)	0.000 (2)
C7	0.048 (3)	0.060 (3)	0.050 (3)	0.015 (2)	0.002 (2)	−0.001 (2)
C8	0.049 (3)	0.064 (3)	0.049 (3)	0.021 (2)	−0.003 (2)	−0.004 (2)
C9	0.049 (3)	0.058 (3)	0.050 (3)	0.018 (2)	−0.004 (2)	−0.006 (2)
C10	0.049 (3)	0.059 (3)	0.052 (3)	0.016 (2)	−0.002 (2)	−0.003 (2)
C11	0.049 (3)	0.066 (3)	0.042 (3)	0.020 (2)	−0.003 (2)	−0.006 (2)
C12	0.048 (3)	0.058 (3)	0.047 (3)	0.017 (2)	−0.004 (2)	−0.001 (2)
C13	0.049 (3)	0.059 (3)	0.041 (3)	0.016 (2)	−0.001 (2)	−0.001 (2)
C14	0.051 (3)	0.067 (3)	0.048 (3)	0.021 (2)	−0.005 (2)	−0.004 (2)
C15	0.042 (3)	0.062 (3)	0.054 (3)	−0.003 (2)	0.002 (2)	0.010 (3)
C16	0.046 (3)	0.063 (3)	0.049 (3)	0.004 (2)	0.005 (2)	0.006 (2)
C17	0.045 (3)	0.058 (3)	0.048 (3)	0.004 (2)	−0.002 (2)	0.012 (2)
C18	0.059 (3)	0.060 (3)	0.060 (4)	−0.004 (3)	−0.016 (3)	−0.002 (3)

### Geometric parameters (Å, °)

**Table:**

OW—HWA	0.8501	C7—H7B	0.9700
OW—HWB	0.8500	C8—C9	1.523 (7)
N1—C17	1.322 (6)	C8—H8A	0.9700
N1—C15	1.370 (6)	C8—H8B	0.9700
N1—C14	1.472 (6)	C9—C10	1.503 (7)
C1—C2	1.512 (8)	C9—H9A	0.9700
C1—H1A	0.9600	C9—H9B	0.9700
C1—H1B	0.9600	C10—C11	1.515 (7)
C1—H1C	0.9600	C10—H10A	0.9700
N2—C17	1.313 (6)	C10—H10B	0.9700
N2—C16	1.363 (6)	C11—C12	1.518 (7)
N2—C18	1.474 (6)	C11—H11A	0.9700
C2—C3	1.507 (7)	C11—H11B	0.9700
C2—H2A	0.9700	C12—C13	1.509 (6)
C2—H2B	0.9700	C12—H12A	0.9700
C3—C4	1.513 (7)	C12—H12B	0.9700
C3—H3A	0.9700	C13—C14	1.498 (7)
C3—H3B	0.9700	C13—H13A	0.9700
C4—C5	1.515 (7)	C13—H13B	0.9700
C4—H4A	0.9700	C14—H14A	0.9700
C4—H4B	0.9700	C14—H14B	0.9700
C5—C6	1.514 (7)	C15—C16	1.352 (7)
C5—H5A	0.9700	C15—H15A	0.9300
C5—H5B	0.9700	C16—H16A	0.9300
C6—C7	1.520 (7)	C17—H17A	0.9300
C6—H6A	0.9700	C18—H18A	0.9600
C6—H6B	0.9700	C18—H18B	0.9600
C7—C8	1.513 (7)	C18—H18C	0.9600
C7—H7A	0.9700		
			
HWA—OW—HWB	107.3	C9—C8—H8B	108.6
C17—N1—C15	108.2 (4)	H8A—C8—H8B	107.6
C17—N1—C14	125.6 (4)	C10—C9—C8	113.9 (4)
C15—N1—C14	126.2 (4)	C10—C9—H9A	108.8
C2—C1—H1A	109.5	C8—C9—H9A	108.8
C2—C1—H1B	109.5	C10—C9—H9B	108.8
H1A—C1—H1B	109.5	C8—C9—H9B	108.8
C2—C1—H1C	109.5	H9A—C9—H9B	107.7
H1A—C1—H1C	109.5	C9—C10—C11	114.7 (4)
H1B—C1—H1C	109.5	C9—C10—H10A	108.6
C17—N2—C16	109.3 (4)	C11—C10—H10A	108.6
C17—N2—C18	125.6 (4)	C9—C10—H10B	108.6
C16—N2—C18	125.1 (4)	C11—C10—H10B	108.6
C3—C2—C1	114.6 (5)	H10A—C10—H10B	107.6
C3—C2—H2A	108.6	C10—C11—C12	114.0 (4)
C1—C2—H2A	108.6	C10—C11—H11A	108.7
C3—C2—H2B	108.6	C12—C11—H11A	108.7
C1—C2—H2B	108.6	C10—C11—H11B	108.7
H2A—C2—H2B	107.6	C12—C11—H11B	108.7
C2—C3—C4	115.0 (4)	H11A—C11—H11B	107.6
C2—C3—H3A	108.5	C13—C12—C11	114.8 (4)
C4—C3—H3A	108.5	C13—C12—H12A	108.6
C2—C3—H3B	108.5	C11—C12—H12A	108.6
C4—C3—H3B	108.5	C13—C12—H12B	108.6
H3A—C3—H3B	107.5	C11—C12—H12B	108.6
C3—C4—C5	114.5 (4)	H12A—C12—H12B	107.5
C3—C4—H4A	108.6	C14—C13—C12	110.7 (4)
C5—C4—H4A	108.6	C14—C13—H13A	109.5
C3—C4—H4B	108.6	C12—C13—H13A	109.5
C5—C4—H4B	108.6	C14—C13—H13B	109.5
H4A—C4—H4B	107.6	C12—C13—H13B	109.5
C6—C5—C4	114.4 (4)	H13A—C13—H13B	108.1
C6—C5—H5A	108.7	N1—C14—C13	113.6 (4)
C4—C5—H5A	108.7	N1—C14—H14A	108.8
C6—C5—H5B	108.7	C13—C14—H14A	108.8
C4—C5—H5B	108.7	N1—C14—H14B	108.8
H5A—C5—H5B	107.6	C13—C14—H14B	108.8
C5—C6—C7	114.3 (4)	H14A—C14—H14B	107.7
C5—C6—H6A	108.7	C16—C15—N1	107.2 (4)
C7—C6—H6A	108.7	C16—C15—H15A	126.4
C5—C6—H6B	108.7	N1—C15—H15A	126.4
C7—C6—H6B	108.7	C15—C16—N2	106.4 (4)
H6A—C6—H6B	107.6	C15—C16—H16A	126.8
C8—C7—C6	114.0 (4)	N2—C16—H16A	126.8
C8—C7—H7A	108.7	N2—C17—N1	108.9 (4)
C6—C7—H7A	108.7	N2—C17—H17A	125.6
C8—C7—H7B	108.7	N1—C17—H17A	125.6
C6—C7—H7B	108.7	N2—C18—H18A	109.5
H7A—C7—H7B	107.6	N2—C18—H18B	109.5
C7—C8—C9	114.4 (4)	H18A—C18—H18B	109.5
C7—C8—H8A	108.6	N2—C18—H18C	109.5
C9—C8—H8A	108.6	H18A—C18—H18C	109.5
C7—C8—H8B	108.6	H18B—C18—H18C	109.5
			
C1—C2—C3—C4	−179.4 (5)	C15—N1—C14—C13	56.5 (6)
C2—C3—C4—C5	179.5 (5)	C12—C13—C14—N1	177.0 (4)
C3—C4—C5—C6	179.2 (4)	C17—N1—C15—C16	−0.9 (6)
C4—C5—C6—C7	−179.8 (4)	C14—N1—C15—C16	176.8 (4)
C5—C6—C7—C8	−178.9 (4)	N1—C15—C16—N2	0.5 (6)
C6—C7—C8—C9	−179.5 (4)	C17—N2—C16—C15	0.2 (6)
C7—C8—C9—C10	179.6 (4)	C18—N2—C16—C15	179.7 (5)
C8—C9—C10—C11	−179.9 (4)	C16—N2—C17—N1	−0.8 (5)
C9—C10—C11—C12	−179.4 (4)	C18—N2—C17—N1	179.7 (4)
C10—C11—C12—C13	178.4 (4)	C15—N1—C17—N2	1.1 (5)
C11—C12—C13—C14	179.6 (4)	C14—N1—C17—N2	−176.7 (4)
C17—N1—C14—C13	−126.2 (5)		

### Hydrogen-bond geometry (Å, °)

**Table:**

*D*—H···*A*	*D*—H	H···*A*	*D*···*A*	*D*—H···*A*
OW—HWA···Br	0.85	2.57	3.397 (5)	165
OW—HWB···Br^i^	0.85	2.61	3.434 (5)	163
C15—H15A···Br^ii^	0.93	2.75	3.659 (5)	166
C17—H17A···OW	0.93	2.36	3.217 (7)	153

Symmetry codes: (i) *x*+1, *y*, *z*; (ii) *x*−1, *y*−1, *z*.
